# Altered Integrative and Conjugative Elements (ICEs) in Recent *Vibrio cholerae* O1 Isolated From Cholera Cases, Kolkata, India

**DOI:** 10.3389/fmicb.2019.02072

**Published:** 2019-09-06

**Authors:** Anirban Sarkar, Daichi Morita, Amit Ghosh, Goutam Chowdhury, Asish K. Mukhopadhyay, Keinosuke Okamoto, Thandavarayan Ramamurthy

**Affiliations:** ^1^Division of Bacteriology, National Institute of Cholera and Enteric Diseases, Kolkata, India; ^2^Graduate School of Medicine, Dentistry and Pharmaceutical Sciences, Okayama University, Okayama, Japan; ^3^Collaborative Research Center of Okayama University for Infectious Diseases in India, National Institute of Cholera and Enteric Diseases, Kolkata, India; ^4^Center for Human Microbial Ecology, Translational Health Science and Technology Institute, Faridabad, India

**Keywords:** cholera, *V. cholerae* O1, tetracycline, antimicrobial resistance, multidrug resistance, integrative conjugative element

## Abstract

The self-transferring integrative and conjugative elements (ICEs) are large genomic segments carrying several bacterial adaptive functions including antimicrobial resistance (AMR). SXT/R391 family is one of the ICEs extensively studied in cholera-causing pathogen *Vibrio cholerae*. The genetic characteristics of ICE-SXT/R391 in *V. cholerae* are dynamic and region-specific. These ICEs in *V. cholerae* are strongly correlated with resistance to several antibiotics such as tetracycline, streptomycin and trimethoprim-sulfamethoxazole. We screened *V. cholerae* O1 strains isolated from cholera patients in Kolkata, India from 2008 to 2015 for antibiotic susceptibility and the presence of ICEs, and subsequently sequenced their conserved genes. Resistance to tetracycline, streptomycin and trimethoprim-sulfamethoxazole was detected in strains isolated during 2008–2010 and 2014–2015. The genes encoding resistance to tetracycline (*tetA*), trimethoprim-sulfamethoxazole (*dfrA1* and *sul2*), streptomycin (*strAB*), and chloramphenicol (*floR*) were detected in the ICEs of these strains. There was a decrease in overall drug resistance in *V. cholerae* associated with the ICEs in 2011. DNA sequence analysis also showed that AMR in these strains was conferred mainly by two types of ICEs, i.e., ICE^TET^ (comprising *tetA, strAB, sul2*, and *dfrA1*) and ICE^GEN^ (*floR, strAB, sul2*, and *dfrA1*). Based on the genetic structure, Kolkata strains of *V. cholerae* O1 had distinct genetic traits different from the ICEs reported in other cholera endemic regions. Transfer of AMR was confirmed by conjugation with sodium azide resistant *Escherichia coli* J53. In addition to the acquired resistance to streptomycin and trimethoprim-sulfamethoxazole, the conjugally transferred (CT) *E. coli* J53 with ICE showed higher resistance to chloramphenicol and tetracycline than the donor *V. cholerae.* Pulsed-field gel electrophoresis (PFGE) based clonal analysis revealed that the *V. cholerae* strains could be grouped based on their ICEs and AMR patterns. Our findings demonstrate the epidemiological importance of ICEs and their role in the emergence of multidrug resistance (MDR) in El Tor vibrios.

## Introduction

The Gram-negative pathogen *Vibiro cholerae* O1 has caused seven pandemics in the history of cholera and tends to cause several epidemics in developing countries (Lekshmi et al., 2018). This pathogen has more than 200 serogroups, but only the serogroups O1 and O139 are associated with epidemic cholera (Lekshmi et al., 2018). The ongoing seventh pandemic is linked with the El Tor biotype of serogroup O1 that has spread in the cholera endemic regions of the world (Lekshmi et al., 2018). The emergence and spread of antimicrobial resistant (AMR) *V. cholerae*, especially those resistant to nalidixic acid, tetracycline, and trimethoprim-sulfamethoxazole, has been reported since the 1980s (Ghosh and Ramamurthy, 2011). Resistance to these antimicrobials has been strongly associated with the presence of integrative and conjugative elements (ICEs) of the SXT/R391 family and its discovery has greatly changed the understanding of AMR in *V. cholerae*.

SXT/R391 ICEs have been characterized/classified based on the conserved core genes, and their integration into the 5′-end of the *prfC* gene that encodes peptide chain release factor 3 (Hochhut and Waldor, 1999). More than 1000 ICEs have been updated in the ICEberg database^1^. Mobility of SXT/R391 ICEs occurs between bacteria by conjugation, resulting in the transfer of several functions including AMR, resistance to heavy metals, regulation of motility and biofilm formation (Waldor et al., 1996; Bordeleau et al., 2010). Five insertion hotspots (H1 to H5) and four variable regions (VRI to VRIV) are also carried by the ICEs (Wozniak et al., 2009). The structure of ICEs changes periodically contributing to the differences in AMR profiles of *V. cholerae.* More than 50 ICEs have been grouped within the SXT/R391 family, of which 30 are reported in clinical and environmental *V. cholerae* strains (Pande et al., 2012). Between 1992 and 2001, 15 ICEs were identified in India and Bangladesh, of which six (SXT^MO10^, ICE*Vch*Ind4, ICE*Vch*Ban5, ICE*Vch*Ban10, ICE*Vch*Ban9, and ICE*Vch*Ind5) were completely sequenced and annotated (Ceccarelli et al., 2011).

Tetracycline has been the drug of choice in treating cholera cases for a long time (World Health Organization [WHO], 2005). A sudden upsurge in the tetracycline resistance (Tet^R^), from 1% in 2004 to 76% in 2007, was reported among *V. cholerae* in Kolkata and it decreased to about 50% in 2009 (Bhattacharya et al., 2011). Similar trends have been observed previously in large cholera epidemics in Tanzania and Madagascar due to extensive prophylactic use of tetracycline (Mhalu et al., 1979; Dromigny et al., 2002). Only a few studies have been carried out to understand the mechanisms of AMR due to ICEs in India (Roychowdhury et al., 2008; Bhattacharya et al., 2011; Kutar et al., 2013). In this study, we screened the AMR patterns of *V. cholerae* O1 Ogawa strains isolated from cholera patients in Kolkata, India from 2008 to 2015 and examined the type of ICEs present by analyzing their backbone genes. Our study revealed the differences between the sequence types of ICEs and recent changes in AMR patterns of *V. cholerae*.

## Materials and Methods

### Clinical Specimens and Bacterial Strains

Stool specimens were collected from the Infectious Diseases Hospital (IDH) and B. C. Roy Children Hospital (BCH), Kolkata, before the patients were treated with antibiotics. Clinical symptoms of diarrheal patients included loose/watery stools with or without dehydration, abdominal cramps, vomiting and fever. Dysentery patients had frequent passage of stool with blood/mucus and mild to severe abdominal pain. For the isolation of *V. cholerae*, all the stool specimens/rectal swabs were enriched in alkaline peptone water (pH 8.0) (Difco, Sparks, MD, United States) for 6 h, followed by inoculation and overnight incubation in thiosulphate citrate bile-salts sucrose agar (TCBS, Eiken, Tokyo, Japan) plates. Sucrose-positive strains were confirmed serologically using commercially available *V. cholerae* O1 poly and monovalent antisera (Denka-Seiken, Tokyo, Japan). To obtain the AMR pattern from 2008 to 2015, 546 out of 1591 strains were randomly selected covering each month of the study period. Sodium azide resistant (Az^R^) *Escherichia coli* J53 (Martínez-Martínez et al., 1998) was used for the conjugation experiments. All the strains were preserved in Luria Bertani (LB) broth (Difco) containing 15% glycerol at −80°C. *E. coli* ATCC 25922 (Clinical and Laboratory Standards Institute [CLSI], 2014) was used as a control strain in antimicrobial susceptibility testing.

### Antibiotic Susceptibility Testing

Susceptibilities of *V. cholerae* strains to ampicillin (AMP, 10 μg), ceftriaxone (CRO, 30 μg), chloramphenicol (CHL, 30 μg), nalidixic acid (NA, 30 μg), ciprofloxacin (CIP, 5 μg), ofloxacin (OFX, 5 μg), norfloxacin (NOR, 10 μg), imipenem (IPM, 10 μg), streptomycin (STR, 10 μg), azithromycin (AZM, 15 μg), tetracycline (TET, 30 μg), trimethoprim-sulfamethoxazole (SXT, 1.25 and 23.75 μg) and gentamicin (GEN, 10 μg), were determined by Kirby-Bauer disk diffusion technique using commercial disks (BD, Sparks, MD, United States) as per the Clinical and Laboratory Standards Institute guidelines (Clinical and Laboratory Standards Institute [CLSI], 2014, 2015).

### Detection of Antibiotic Resistance Encoding Genes

Total nucleic acid of *V. cholerae* strains was extracted using a QIAamp DNA mini kit (Qiagen, Hilden, Germany) following the manufacturer’s instructions. The integrase gene (*int*^*SXT*^) present in ICE was amplified by PCR using previously described primer pair int1-F and int1-B (Dalsgaard et al., 2001). Beside *int*^*S*XT^, PCR was also performed to detect the presence of resistance encoding genes for chloramphenicol (*floR* and *cat*), streptomycin (*strA* and *strB*), and sulfonamide (*sul1* and *sul2*) (Sarkar et al., 2015a). Primer pairs VCtetA.F-(5′- ACGGTATCCTGCTGGCACTGTATG-3′) and VCtetA.R-(5′- CATCCATATCCAGCCATCCCAACT-3′) and VctetR.F-(5′-GA AGTGGGAATGGAAGGGCTGAC-3′) and VctetR.R-(5′-AG CCTCTGTGCCATCATCTTG-3′) were designed to detect the Tet^R^ encoding gene (*tetA*), and the repressor protein (*tetR*) for a regulatory portion of resistance cassettes, respectively. Representative amplicons were purified using a PCR product purification kit (Qiagen) and sequenced using the ABI Big Dye terminator cycle sequencing ready reaction kit, version 3.1 (Applied Biosystems, Foster City, CA, United States) in an automated DNA sequencer (ABI 3730, Applied Biosystems). The sequences were assembled and analyzed using DNASTAR software (DNASTAR Inc., Madison, WI, United States).

### Conjugation

To test the mobility of the ICEs, conjugation assay was carried out using a representative ICE-positive *V. cholerae* O1 strain as donor with *E. coli* J53 (Az^R^, Martínez-Martínez et al., 1998). In brief, overnight cultures of the bacteria were mixed at 1:2 donor-to-recipient ratios in 1 ml of LB broth and allowed to grow overnight at 37°C. The donor and recipient suspensions were diluted serially in phosphate buffer saline (PBS) and plated on TCBS and MacConkey agar plates, respectively, to confirm the purity and count the number of colonies. To detect the conjugally transferred *E. coli* J53 (CT-*E. coli* J53), MacConkey agar supplemented with streptomycin (100 μg/ml) and sodium azide (AZD, 100 μg/ml) was used. Transconjugants were confirmed as ICE-positive by PCR analysis, followed by PCR amplicon sequencing. To confirm the resistance phenotype, antibiotic susceptibility patterns of the donor, recipient and transconjugants were determined after their growth on Mueller-Hinton (MH, Difco) agar by disk diffusion method. An increase in resistance of transconjugants was quantified by determining the MICs of CHL, STR, TET, and SXT using *E*-test strips (AB bioMérieux, Solna, Sweden).

### Pulsed-Field Gel Electrophoresis (PFGE)

Clonal analysis of representative *V. cholerae* O1 strains isolated between 2008 and 2015 was made following the PulseNet protocol (Cooper et al., 2006). *V. cholerae* O1 strains were used after digesting the DNA with *Not*I [New England Biolabs (NEB), Ipswich, MA, United States]. *Xba*I (NEB) digested *Salmonella* Braendruff H-9812 was used as a DNA size marker. The PFGE run conditions were generated by the auto-algorithm mode of the CHEF Mapper system (Bio-Rad, Hercules, CA, United States). PFGE profiles were analyzed by the BioNumerics version 4.0 software (Applied Maths, Sint-Martens-Latem, Belgium) using the Dice coefficient and unweighted pair group method using arithmetic averages (UPGMA).

### Whole Genome Sequence Analysis

The whole genome sequences submitted from our previous study (Imamura et al., 2017) were used in the analysis. The open reading frames (ORFs) from the contigs were generated by contig integrator for sequence assembly (CISA) using Glimmer-MG program^2^. Nucleotide sequences and amino acid sequences were obtained from these ORFs and translated in the appropriate frame. The predicted ORFs were annotated using CANoPI (Contig Annotator Pipeline) that also includes BlastX search for each ORF sequence against the “nr” database of NCBI^3^. From the whole genome sequence data of representative strains (Tet^R^ IDH 1986 and Tet^*S*^ IDH 4268), we have used part of the ICE region in the analysis. The contigs were aligned, assembled and compared with SEQMAN, assembly module of DNASTAR’s LASERGENE with published sequences like ICE*Vch*Ind5 (GQ463142), ICE*Vch*Ban5 (GQ463140), MO10 (AY055428), etc. For confirmation, PCR was performed targeting important short regions of the ICEs (*rumAB, traI, traC, setR, traA-traC*, and *traG*) with previously described primers (Bani et al., 2007). Published ICE sequences were used for homology search. ORF search and gene prediction were performed for the complete ICE region with EditSEQ, Lasergene software (DNASTAR), and pairwise alignment was analyzed by blastN and blastP homology search using the NCBI database.

### Nucleotide Sequence Submission

The AMR encoding gene cassettes and their flanking sequences of representative ICE of Tet^R^ and Tet^*S*^ *V. cholerae* O1 have been submitted in GenBank (Accession numbers MK165649 and MK165650, respectively).

### Ethics and Biosafety Statements

The Ethics and Biosafety Committees of National Institute of Cholera and Enteric Diseases, Kolkata approved this study (A:1/2015-IEC). Each participant/parent in the case of children gave written informed consent. All the experiments were performed following Biosafety Level-2 standards.

## Results

### Prevalence of Cholera

**FIGURE 1:**
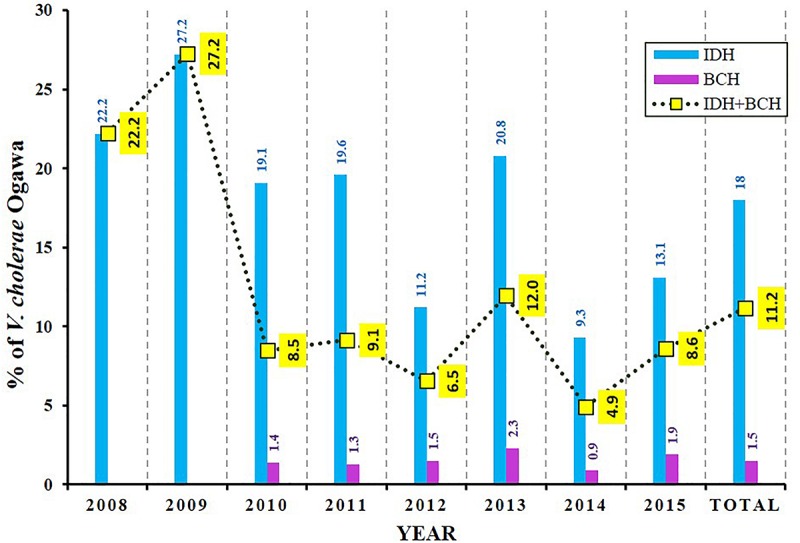
Isolation rate of *V. cholerae* O1 Ogawa among diarrheal patients. BCH sample collection was started from 2010 onward. The dotted line with yellow boxes represent the mean incidence of cholera in IDH and BCH.

During 8 years of surveillance from 2008 to 2015, the isolation rate of *V. cholerae* O1 Ogawa was about 11% (1591 of 14237 tested samples) (Figure 1). The incidence of this pathogen in BCH samples was very low (∼2%) but was found to be much higher (∼18%) in IDH samples. As shown in Figure 1, the mean incidence of cholera in IDH/BCH fluctuated between 4.9% (2014) and 27.2% (2009). Except for children ≤5 years, *V. cholerae* O1 remained one of the important bacterial pathogens. The incidence of *V. cholerae* O1 varies in certain extent from year to year (Figure 1).

### Antimicrobial Resistance

All the *V. cholerae* O1 strains isolated were consistently resistant to NA. Tet^R^ gradually decreased from 58% in 2008 to 48% in 2009, followed by a further drop in 2010 (9%). Thereafter, all the strains isolated between 2011 and 2013 were found to be susceptible to TET (Table 1). Remarkably, Tet^R^ trait increased again in 2015 (56%). There was a marked change in AMP resistance each year with highest in 2010 (94%) and lowest in 2012 (21%) (Table 1). About three fourth of the strains were resistant to AMP in 2009 and 2011 (>76%). Thereafter, most of the *V. cholerae* isolated from 2013 to 2015 were found to be susceptible to AMP.

**TABLE 1:** Resistance of *V. cholerae* O1 Ogawa against different antibiotics.

**Year (n)**	**% of resistance**					
	**TET**	**CHL^∗^**	**STR**	**SXT**	**AMP**	**NA**
2008 (76)	58	33	92	92	53	100
2009 (120)	48	45	96	99	76	100
2010 (53)	9	91	98	98	94	100
2011 (52)	0	25	23	31	77	100
2012 (48)	0	58	67	69	21	100
2013 (87)	0	91	98	99	0	100
2014 (44)	2	86	91	93	0	98
2015 (66)	56	38	94	92	0	100

*^∗^Except three, rest of the strains were intermediate (i) to CHL.*

Throughout the study period, only three *V. cholerae* strains were found to be fully resistant to CHL and the rest of the *floR* containing strains showed intermediate resistance [CHL(i)] to this antibiotic. Interestingly, resistance to TET was found to be inversely proportional to CHL(i), i.e., strains showing Tet^R^ had intermediate resistance to CHL. The CHL(i) trait increased in 2010 (91%) when Tet^R^ was very low (9%) but dropped to 38% with the re-emergence of Tet^R^ in 2015 (56%). Resistance to STR and SXT were detected in most of the *V. cholerae* O1 strains. Resistance to these antimicrobials was >90% from 2008 to 2010 and 2013 to 2015. Interestingly, there was a sudden decrease in STR and SXT resistance (23 and 31%, respectively, in 2011) followed by an increase in 2012 (67 and 69%, respectively) (Table 1).

This study shows the changing profile of MDR in *V. cholerae* from Kolkata; MDR profiles NA-STR-SXT-TET-AMP and NA-STR-SXT-TET were predominant during 2008, 2009 and 2015 (Table 2), while from 2009 to 2010 and 2012 to 2014 the MDR profiles NA-STR-SXT-CHL(i), and NA-STR-SXT-CHL(i)-AMP were found in more than 50% of the *V. cholerae* O1 strains.

**TABLE 2:** Percentage of resistance pattern in *V. cholerae* O1 strains during 8 years in Kolkata.

**Resistance profile/Year**	**2008 (*n* = 76)**		**2009 (*n* = 120)**		**2010 (*n* = 53)**		**2011 (*n* = 52)**		**2012 (*n* = 48)**		**2013 (*n* = 87)**		**2014 (*n* = 44)**		**2015 (*n* = 66)**	
NA-STR-SXT-TET-AMP	27.6	**58**	35.8	**48**	3.8	**9**							0.0	**2**	0.0	**53**
NA-STR-SXT-TET	30.3		12.5		5.7								2.3		52.9	
NA-STR-SXT-CHL(i)-AMP	18.4	**34**	40.0	**51**	88.7	**89**	19.6	**24**	6.4	**72**	0.0	**100**	0.0	**98**	0.0	**46**
NA-STR-SXT-CHL(i)	15.8		10.8				4.3		66.0		100		97.7		45.7	
NA-AMP	6.6	**8**	0.8	**1**	1.9	**2**	56.5	**76**	14.9	**28**					0.0	**1**
NA	1.3		0.0		0.0		19.6		12.8						1.0	

*(i), intermediate resistance for CHL. Numbers in bold represents cumulative percentage of resistance patterns.*

### ICE Comprising Antimicrobial Resistance Genes

**FIGURE 2:**
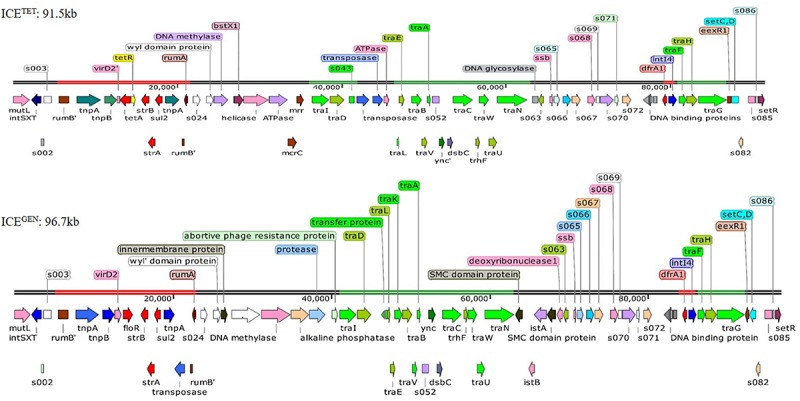
Structure of the two ICEs found in MDR *V. cholerae* O1 Ogawa strains. The AMR genes are shown in red, the genes responsible for the transfer are presented in green, and transposases and integrases are shown in blue. The other shades represented miscellaneous features.

While analyzing the sequences of the resistance gene clusters, two types of ICEs could be detected, i.e., ICE^TET^ (Acc No. MK165649; Tet^R^ IDH 1986) and ICE^GEN^ (Acc No. MK165650; Tet^*S*^ IDH 4268). The superscript “GEN” stands for “general.” Although the ICE^GEN^ was very similar to the ICE*Vch*Ind5 with 99% identity at 100% query coverage, the ICE^TET^ had only 99% identity at 70% query coverage. The structure of these two ICEs with ORFs is shown in Figure 2. The ICE^GEN^ was found to be larger (96.7 kb) than ICE^TET^ (91.5 kb). SXT and STR resistant *V. cholerae* O1 strains were positive for *int*^*SXT*^. Detection of ICEs was >90% in 2008 and 2009, with highest in 2010 (98%), followed by an abrupt decrease in 2011 (23%). However, in 2012, 68% of the *V. cholerae* O1 strains harbored the ICEs. Interestingly, except for NA, the *int*^*SXT*^ negative strains were susceptible to most of the antimicrobials tested in this study. In the 1st type, ICE^TET^ carried a TET efflux pump encoding gene (*tetAR*; *tetA* is a gene encoding TET efflux pump and *tetR* is a repressor protein regulating the *tetA* expression) and in the 2nd type, ICE^GEN^ harbored CHL efflux pump encoding gene (*floR*). ICE^GEN^ has high similarity (99%) with the ICE*Vch*Ind5, the most common ICE detected among seventh-pandemic El Tor vibrios (Spagnoletti et al., 2014; Bioteau et al., 2018). This ICE also has very high similarity to the ICE*Vch*Hai1 from the Haitian *V. cholerae* lineage (Sjölund-Karlsson et al., 2011).

The ICE^GEN^ and ICE^TET^ had *sul2*, *strBA* in the AMR gene cluster conferring resistance to SXT and STR, respectively. Generally, in *V. cholerae*, the presence of *tet* alleles within the ICE gene clusters is uncommon. In the prototype SXT^MO10^, resistance gene cluster comprised *dfr18, floR, strBA, sul2* encoding resistance to trimethoprim, CHL, STR, and sulfamethoxazole, respectively (Table 3). In ICE*Vch*Ind4, there was a major deletion of *dfr18* gene in the cluster. In IDH1986 and IDH14268 strains, a class 4 integron carrying the trimethoprim resistance encoding *dfrA1* was identified in H3 located within the *s073*-*traF* locus. Such arrangement exists in ICE*Vch*Ind5 backbone (Figure 2) and ICE*Vch*Ind1. But, *tetA* gene was absent in these ICEs.

**TABLE 3:** Comparison of the ICE gene cluster with the other SXT/R391 ICE family members.

**ICE**	**Host strain**	**Country and year of isolation**	**Size (bp)**	**Resistance gene content**	**GenBank accession number**	**References**
ICE*Vch*Mex1	*Vibrio cholerae* non O1-O139	Mexico 2001	82839	*–*	GQ463143	Burrus et al., 2006
**ICE^TET^**	*Vibrio cholerae* O1 (IDH1986)	India 2009	91463	*tetAR, strBA, sul2, dfrA1*	MK165649	In this study
**ICE^GEN^**	*Vibrio cholerae* O1 (IDH4268)	India 2012	96718	*floR, strBA, sul2, dfrA1*	MK165650	In this study
ICE*Vch*Ind4	*Vibrio cholerae* O139	India 1997	95491	*floR, strBA, sul2*	GQ463141	Wozniak et al., 2009
ICE*Vch*Ind5	*Vibrio cholerae* O1	India 1994	97847	*floR, strBA, sul2, dfrA1*	GQ463142	Ceccarelli et al., 2011
ICE*Vch*Ban5	*Vibrio cholerae* O1	Bangladesh 1998	102131	*floR, strBA, sul2, dfrA1*	GQ463140	Wozniak et al., 2009
ICE*Pal*Ban1	*Providencia alcalifaciens*	Bangladesh 1999	96586	*floR, strBA, sul2, dfrA1*	GQ463139	Wozniak et al., 2009
ICE*Vfl*Ind1	*Vibrio fluvialis*	India 2002	91369	*dfr18, floR, strBA, sul2*	GQ463144	Wozniak et al., 2009
ICE*Vch*Moz10/ICE*Vch*B33	*Vibrio cholerae* O1	Mozambique 2004	104495	*floR, strBA, sul2, tetA’*	ACHZ00000000	Taviani et al., 2009
ICE*Pmi*Usa1	*Proteus mirabilis*	United States 1986	79733	*–*	AM942759	Pearson et al., 2008
ICE*Vch*Ban9	*Vibrio cholerae* O1	Bangladesh 1994	106124	*floR, strBA, sul2, dfrA1, tetA’*	CP001485	Wozniak et al., 2009
ICE*Vch*Ban8	*Vibrio cholerae* non O1-O139	Bangladesh 2001	105790	*–*	NZ_AAUU00000000	Wozniak et al., 2009
SXT^MO10^	*Vibrio cholerae* O139	India 2002	99452	*dfr18, floR, strBA, sul2*	AY055428	Beaber et al., 2002
R391	*Providencia rettgeri*	South Africa 1967	88532	*kanR, merRTPCA*	AY090559	Böltner et al., 2002
ICE*Pda*Spa1	*Photobacterium damselae*	Spain 2003	102985	*tetAR*	AJ870986	Juíz-Río et al., 2005
ICE*Spu*PO1	*Shewanella putrefaciens*	Pacific Ocean 2000	108623	–	CP000503	Wozniak et al., 2009

Detection of ICE^TET^ in *V. cholerae* O1 decreased from 2008 (58% Tet^R^) to 2010 (9% Tet^R^). All the *V. cholerae* O1 strains isolated during 2011–2013 lacked ICE^TET^. In 2015, however, the *tetAR* was again detected in a higher number of strains (56% Tet^R^). In contrast, ICE^GEN^ was detected throughout the study period. AMR gene cassettes located within the *rumB* locus are also different. From 2011 to 2013, the *tetAR* locus in ICE^TET^ was replaced by *floR* gene of ICE^GEN^. This feature marked the difference of ICE^TET^ from ICE*Vch*Lao1, where *floR* and *tetA* were concurrently present.

Based on the presence of the AMR encoding genes harbored by these elements, the genetic background of ICE^TET^ appears to be very different from the other ICEs carrying the *tet*. The ICE*Pda*Spa1 was found to have only the TET resistance determinant located within *rumBA* operon (Table 3). Whereas, in the ICE*Vch*Lao1, resistance genes of CHL (*floR*), STR (*strBA*) and sulfamethoxazole (*sul2*) were present along with *tetA*. But, the ICE*Vch*Lao1 did not carry *dfrA1* or *dfr18* that confer resistance to trimethoprim in SXT^*ET*^ and SXT^MO10^, respectively. Within the resistance gene cluster of 2008–2010 strains of *V. cholerae* in Kolkata, a deletion of *floR* gene, which was present upstream of the *tetA* gene in ICE*Vch*Lao1 and ICE*Vch*Ban9 was detected.

### Genetic Structure of the ICEs

Generally, the genetic organization of ICE^TET^ and ICE^GEN^ was similar to that of the other members of this family. Many ORFs were commonly shared by these ICEs; most of them being in the conserved core genes (Beaber et al., 2002). Five conserved insertion hotspots are located between *s043 (traJ)* and *traL* (H1), *traA* and *s054* (H2), *s073* and *traF* (H3), *traN* and *s063* (H4), and *s025* and *traID* (H5) (Wozniak et al., 2009).

Five ORFs were found in the H1 of ICE^TET^ that include *tbp* (integrase catalytic subunit), a hypothetical protein (HP), transposase, ISPsy4 transposition helper protein and DNA helicase family protein. These ORFs present in H1 are unique compared to other reported ICEs. Instead of *mosA*, *mosT* that encode toxin-antitoxin reported in the H2 of other ICEs, the ICE^GEN^ and ICE^TET^ have 3 ORFs with *ynd* (transcriptional regulator with AbiEi antitoxin N-terminal domain), *ync* (nucleotidyl transferase AbiEii/AbiGii toxin family protein) and *dsbC* (disulfide isomerase DsbC). H3 of ICE^GEN^ and ICE^TET^ contains 7 ORFs with *bleR* (glyoxalase/bleomycin resistance), *araC* (AraC family transcriptional regulator; helix-turn-helix domain protein), a hypothetical protein, XRE family transcriptional regulators, a putative membrane protein, *dfrA1* (trimethoprim-resistance) and *intI4* (site-specific recombinase IntI4). Of these, AraC, XRE, and DFRA1 were reported in ICE*Vch*Moz10. H3 in ICE^GEN^ and ICE^TET^ is varied from ICE*Vch*Ind4, SXT^MO10^, ICE^R391^ ICE*Vch*Mex1, ICE*Vfl*Ind1, ICE*Pmi*USA1, ICE*Spu*PO1 (Wozniak et al., 2009). H4 of ICE^TET^ was small with 2 ORFs, whereas the ICE^GEN^ had 5 ORFs with two SMC (structural maintenance of chromosome) domain proteins, *istB* (ATP binding domain), *istA* (integrase catalytic subunit) and deoxyribonuclease I. The ORF content of H4 in these ICEs is different from the others. In ICE^GEN^ and ICE^TET^, the H5 has 10–11 gene combinations with the new ORFs of WYL domain protein, N-6 DNA methylase, restriction endonuclease subunit S, *Bst*XI (restriction endonuclease protein), ATPases associated with diverse cellular activities (AAA) family protein, McrC (putative protein) in ICE^TET^ and WYL domain-containing protein with three conserved amino acids, BrxC (BREX system P-loop protein), PglX (BREX-1 system adenine-specific DNA-methyltransferase) and abortive phage resistance protein in ICE^GEN^. These changes in the hotspot regions may not have an obvious effect on the ICE, as they did not influence its transfer. VR-II has an insertion of single ORF, *mutL* similar to the ICE contigs circulating in India and Bangladesh. In the VRIII of ICE^TET^, 12 ORFs [Tn3 (transposase), *tnpA* (transposase), *tnpB* (InsA transposase), truncated *virD2*, *tetA*, *tetR*, IS91 transposase, *strB*, *strA*, *sul2*, *tnpA* tn3 transposase, *s021*] were identified within the two *rumB* portions. In the case of ICE^GEN^, 14 ORFs [Tn3 (trnansposase), *tnpA* (transposase), *tnpB* (InsA transposase), *virD2* (relaxase), *floR*, LysR family protein, truncated transpoase, *strB*, *strA*, *sul2*, *tnpA* tn3 transposase, truncated *s021*, putative transpoase, truncated *mutL*] have been detected.

The restriction-modification system is composed of genes encoding the functions of DNA modification, recombination, and repair (Wozniak and Waldor, 2009). ICE^GEN^ and ICE^TET^ were found to have a type I restriction-modification system in the H5. In the ICE backbones, there were sequences in the ORFs located between *s024* and *traI* in Kolkata strains (Figure 2). In ICE^GEN^ carrying strains, after the *traN* locus, there was an insertion of *istBA* gene flanked by gene encoding SMC domain protein. This arrangement was not observed in *V. cholerae* strains with ICE^TET^. Though these two types of ICEs had same *traFHG* locus, ORFs encoding transposases and ATPase were found incorporated between the *traD* and *traE* locus only in ICE^TET^. In contrast, the ICE^GEN^ possessed an intact transfer region (Figure 2). In ICE*Vch*Ind4, there was a major deletion of *dfr18* gene in the cluster. In strains with ICE^GEN^ or ICE^TET^, a class 4 integron carrying the trimethoprim resistance encoding *dfrA1* was identified in the H3 region located within the *s073*-*traF* locus. Similar gene configuration exists in the ICE*Vch*Ind1 and ICE*Vch*Ind5 backbones. In the 2008–2010 strains of *V. cholerae* in Kolkata, Tet^R^ in ICE was primarily due to *tetA*, whose presence was previously reported in ICE*Pda*Spa1 of *Photobacterium damselae*, ICE*Vch*Lao1 and ICE*Vch*Ban9 of *V. cholerae* O1 from Laos and Bangladesh, respectively (Table 3).

The *tra* loci appeared to be derived from a common ancestor and were mostly present in ICEs of *V. cholerae* strains. These loci are crucial for the transfer of ICEs and generating the conjugation machinery (Wozniak et al., 2009). Similar to the other ICEs backbone, the *tra* genes are arranged in four clusters in IDH1986 and IDH4268 strains, spanning more than 25 kb. Cluster 1 contains the genes and sequences necessary for transfer initiation, the nickase (encoded by *traI*), and the coupling protein (encoded in the *traD*). The mating pair formation function is controlled by three gene clusters: (i) *traLEKBVA*, (ii) *traC/trhF/traWUN*, and (iii) *traFHG* (Figure 2).

### Comparison of Conserved Genes in the ICEs

**FIGURE 3:**
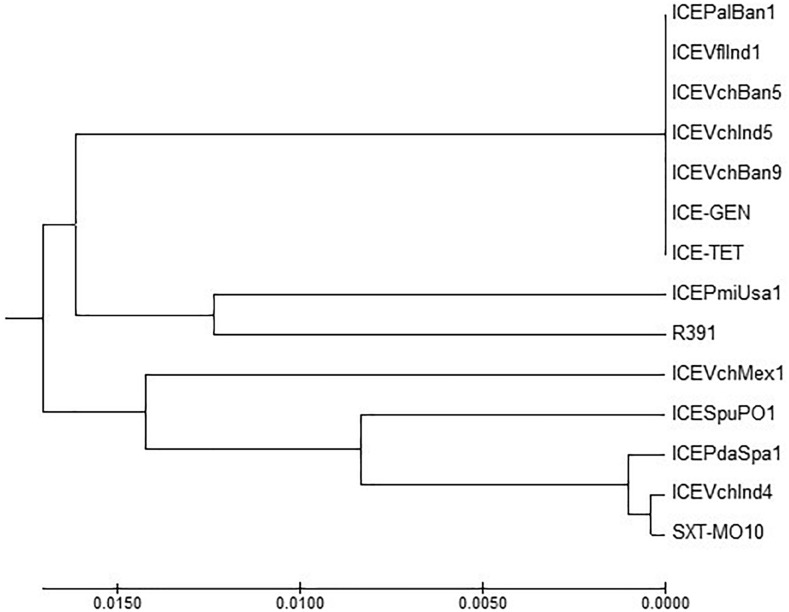
MEGA7 analysis based (Kumar et al., 2016) evolutionary relationships of taxa of *int* of *V. cholerae* O1 strains.

**FIGURE 4:**
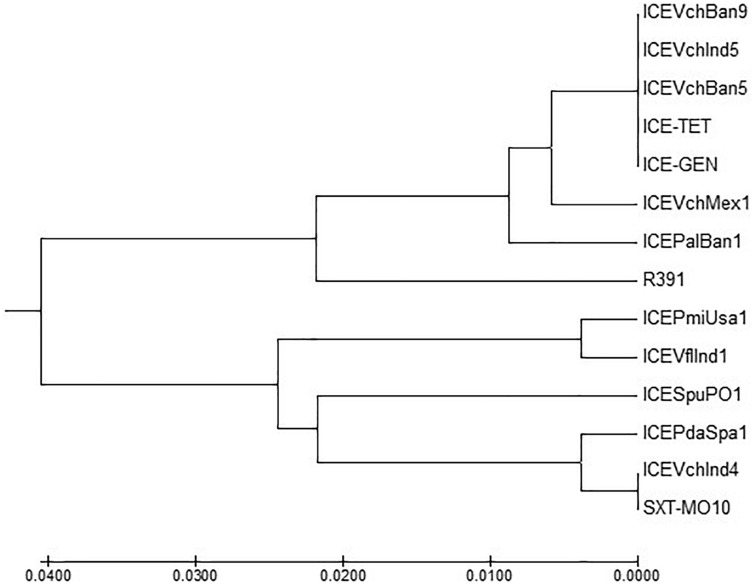
MEGA7 analysis based (Kumar et al., 2016) evolutionary relationships of taxa of *eex* of *V. cholerae* O1 strains.

**FIGURE 5:**
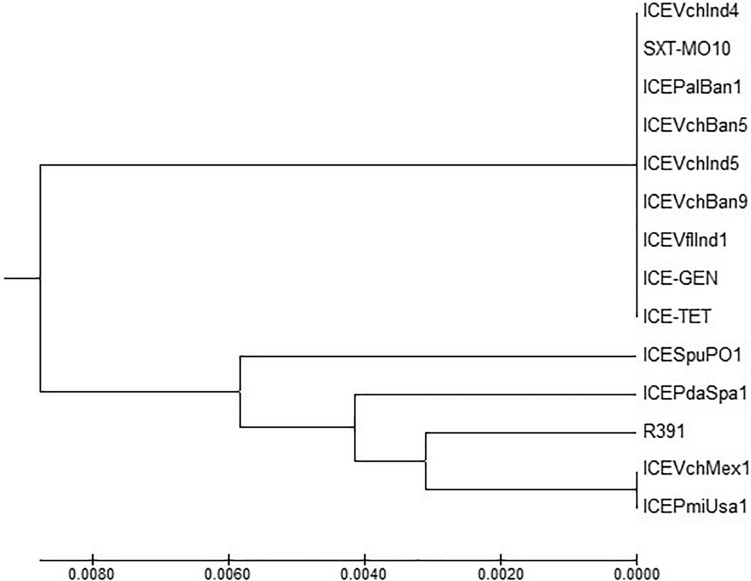
MEGA7 analysis based (Kumar et al., 2016) evolutionary relationships of taxa of *setR* of *V. cholerae* O1 strains.

ICE^TET^ and ICE^GEN^ shared the same exclusion group (EexR). This EexR system might have been transferred from R391 type ICEs (Marrero and Waldor, 2007). The site-specific integration of the ICE is mediated through integrase enzyme encoded in the *int*. The *int* of ICE^TET^ and ICE^GEN^ harboring *V. cholerae* O1 is identical to those present in the strains that have ICE*Pal*Ban1 of *P. alcalifaciens*, ICE*Vf*Ind1 of *V. fluvialis* and ICE*Vch*Ban5, ICE*Vch*Ban9 and ICE*Vch*Ind5 of *V. cholerae* (Figure 3). These ICEs are distinct from those reported in *Proteus mirabilis*, *Providencia rettgeri, Shewanella putrefaciens*, *P. damselae* as well as in other *V. cholerae* with ICE*Vch*Mex1, ICE*Vch*Ind4, and SXT^MO10^. SetR and SetC/D are the key regulators of ICEs, which are closely followed by the genes encoding for inner membrane proteins (Eex and TraG) of the donor and recipient cells. Eex and TraG facilitate entry-exclusion in the SXT/R391 family of ICEs. In the cluster tree, *eex* genes of the ICE^TET^ and ICE^GEN^ showed high homology with ICE identified in ICE*Vch*Ban5, ICE*Vch*Ban9, ICE*Vch*Ind5, but was distantly related to other ICEs of *V. cholerae* and other species (Figure 4). *setR* in the ICE^TET^ and ICE^GEN^ are identical with that in ICE*Vch*Ind4, ICE*Vch*Ind5, ICE*Vch*Ban5, ICE*Vch*Ban9, SXT^MO10^, ICE*Vf*Ind1, ICE*Pal*Ban1 but different from ICE*Vch*Mex1 and ICEs of other species (Figure 5).

### Transfer of ICEs

To test the transferability of the *V. cholerae* ICEs, we selected ICE^TET^ and ICE^GEN^ carrying strains (IDH1986 and IDH1439, respectively). Both the types of ICEs could be transferred to *E. coli* J53 by conjugation. The transconjugants acquired additional resistance against SXT and STR (Table 4). Remarkably, CT-*E. coli* J53 from ICE^GEN^ was highly resistant to CHL compared to the donor *V. cholerae* O1 strain, which showed reduced susceptibility to this antibiotic. Similarly, CT-*E. coli* J53 from ICE^TET^ expressed more resistance against TET than the donor *Vibrio* (Table 4). The frequency of transfer ranged from 3 × 10^–5^ to 5 × 10^–6^ transconjugants/recipient.

**TABLE 4:** Increased resistance attributed by acquired ICE in transconjugants.

**Strain**	**Resistance profile**	**MIC value (μg/ml)**			
		**SXT**	**STR**	**TET**	**CHL**
IDH1986 (*V. cholerae* O1 Ogawa)	NA-TET-SXT-STR	>32	192	16	1
CT-*E. coli* J53/ICE^TET^ (Transconjugant)	TET-SXT-STR-AZD	>32 (>600 fold)	48 (24 fold)	24 (48 fold)^∗^	3
*E. coli* J53 (Recipient)	AZD	0.047	2	0.5	3
CT-*E. coli* J53/ICE^GEN^ (Transconjugant)	CHL-SXT-STR-AZD	>32 (>600 fold)	64 (32 fold)	0.5	>256 (>85 fold)
IDH1439 (*V. cholerae* O1 Ogawa)	NA-SXT-STR-{CHL(i)}	>32	128	0.5	8

*^∗^Increase in fold compared to the recipient.*

### PFGE Analysis

**FIGURE 6:**
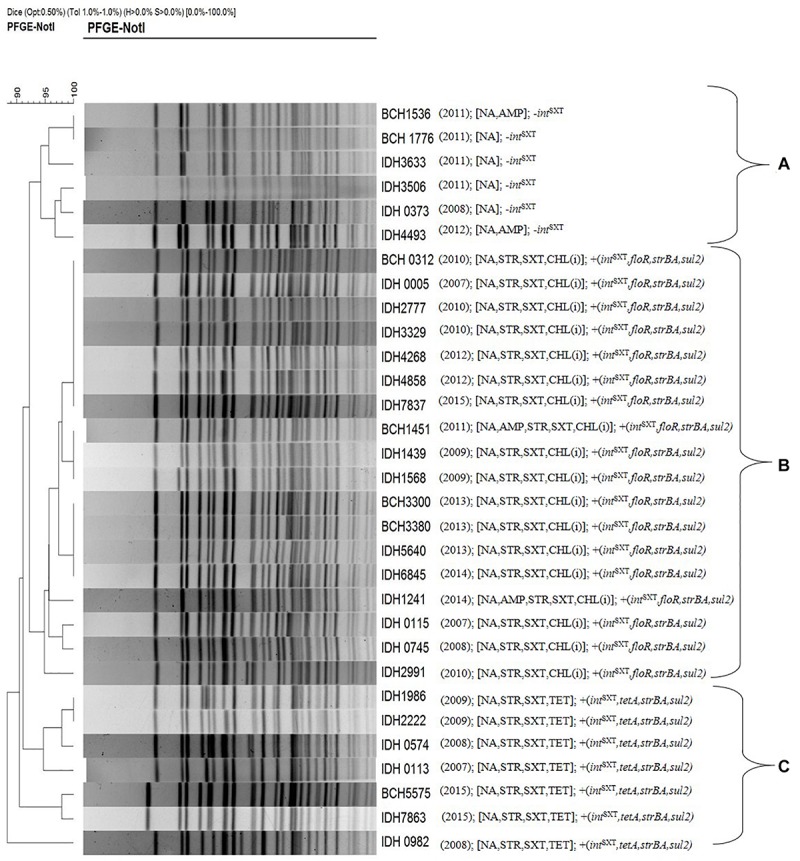
PFGE profile of the representative *V. cholerae* O1 strains with their antimicrobial resistance. Cluster **A**, strains devoid of *int*^*SXT*^ or ICE; Cluster **B,** strains carrying ICE^GEN^; Cluster **C,** strains having ICE^TET^. Number in the parenthesis represents the year of isolation.

Pulsed-field gel electrophoresis was performed to identify the clonal relationship between ICE^TET^ and ICE^GEN^ carrying *V. choleare* strains. It was found that the *V. cholerae* O1 strains displayed clonal clusters reflecting their MDR profile, which indirectly revealed the composition of AMR encoding genes in the ICEs (Figure 6). Cluster A represented *Vibrio* strains devoid of the ICEs. These strains were only resistant to NA. Strains with ICE^GEN^ were present in cluster B. These strains are resistant to NA, SXT and exhibited intermediate susceptibility to CHL. Cluster C contained the ICE^TET^ harboring strains that showed resistance to NA, SXT, and TET (Figure 6).

## Discussion

Cholera is endemic in the Indian subcontinent and it has spread to several other parts of the world (Mutreja et al., 2011). In Kolkata, MDR *V. cholerae* is associated with sporadic cholera for many years (Garg et al., 2000; Nair et al., 2010). *V. cholerae* O1 was susceptible to several antibiotics before 1980s, but developed resistance to SXT in the following years (Ghosh and Ramamurthy, 2011). *V. cholerae* O1 El Tor biotype that re-emerged in 1994 may have acquired SXT resistance phenotype from the O139 serogroup (Ramamurthy et al., 2003). Investigations conducted almost during the same period in several cholera endemic regions in India showed that the isolation rate of *V. cholerae* O1 was lesser than Kolkata, but the AMR pattern followed nearly the same trend, especially to tetracycline (Taneja et al., 2010; Das et al., 2011; Bhattacharya et al., 2012; Borkakoty et al., 2012; Mandal et al., 2012; Roy et al., 2012; Palewar et al., 2015; Bhuyan et al., 2016; Jain et al., 2016; Torane et al., 2016; Pal et al., 2018).

From 2010 to 2012, *V. cholerae* strains with AMR profiles of NA-STR-SXT-TET-AMP and NA-STR-SXT-TET were completely replaced with NA-STR-SXT-CHL(i)-AMP and NA-STR-SXT-CHL(i) along with NA-AMP and NA. Strains with the AMR profile of NA-STR-SXT-TET appeared again in 2015 (53%). Though the number of *V. cholerae* strains with the NA-SXT-STR-CHL(i) profile was highest from 2013 to 2014 (98–100%), it has reached to 46% with the re-emergence of Tet^R^ in 2015. The appearance of Tet^R^ in *V. cholerae* O1 Ogawa in 2008 has been reported from northern parts of India (Taneja et al., 2010). Tet^R^ has been previously reported mostly in Inaba serotype (Jesudason, 2006; Roychowdhury et al., 2008). Presence of *tetA, floR, strBA, sul2, dfrA1* within the AMR gene cassettes has positive correlation with the phenotypic expression of drug resistance against TET, CHL, STR, and SXT (Dalsgaard et al., 2001; Hochhut et al., 2001; Wang et al., 2016). It is interesting to note that although *dfrA18* conferring resistance to trimethoprim was reported in MO10, later it was replaced by the *dfrA1* allele in a class IV integron located in the H3 (Wozniak et al., 2009).

In our study, *floR* and *tetA* genes were not found to coexist within the VRIII present in the *rumB* locus. Previous reports, however, had shown the presence of both *floR* and *tetA* in the *V. cholerae* ICE*Vch*Lao1 isolated from the Laos, ICE*Vch*B33 from Beira, Mozambique (Iwanaga et al., 2004; Taviani et al., 2009). Depending upon the presence of resistance cassettes in the ICEs, we found two types of ICEs in our study namely ICE^GEN^ and ICE^TET^. Though the ICE backbone of ICE^GEN^ was similar to those of SXT^MO10^ and SXT^*ET*^, it had 99% structural similarity to ICE*Vch*Ind5. Lineages of ICE*Vch*Ind5 of *V. cholerae* O1 strains causing epidemics in the Indian subcontinent might have spread to Africa (Valia et al., 2013).

ICE^GEN^ circulating in *V. cholerae* strains from Kolkata belonged to the group 1 ICE, which comprised ICE*Vch*Ind5 (India, 1994–2005), ICE*Vch*Ban5 (Bangladesh, 1998), ICE*Vch*Hai1 (Haiti, 2010), ICE*Vch*Nig1 (Nigeria, 2010), and ICE*Vch*Nep1 (Nepal, 1994) (Marin et al., 2014). Type I restriction-modification system systems of ICE^GEN^ and ICE^TET^ were also reported in the other ICEs families, such as ICE*Vch*Mex1 and ICE*Spu*PO1 (Burrus et al., 2006; Pembroke and Piterina, 2006). ICEs are constantly spreading in different geographical areas. ICE*Vch*B33, which is different from other ICEs of SXT/R391 was first identified in *V. cholerae* O1 strains from India in 1994 and then Mozambique in 2004 (Taviani et al., 2009). Similar to *V. cholerae* O1 from India with ICE*Vch*Ind1, the other ICEs identified in Vietnam, Laos, and Mozambique (ICE*Vch*Vie1, ICE*Vch*Lao1, and ICE*Vch*B33, respectively) lack the trimethoprim resistance encoding *dfr18*, but carried *virD2* and *floR*, conferring resistance to CHL (Taviani et al., 2009). Majority of the *V. cholerae* O1 isolated in Kolkata from 1989 to 1990 had STX^MO10^/ICE*Vch*Ind4. This ICE was replaced by ICE*Vch*Ind5/ICE*Vch*Ban5 in the subsequent years (Weill et al., 2017, 2019).

In this study, the ICE^TET^ detected in *V. cholerae* O1 strains had significant structural dissimilarities with ICE*Vch*Ban9 (Bangladesh, 1994), ICE*Vch*Moz10 (Mozambique, 2004), ICE*Vch*B33 (Beira, 2004), and ICE*Vch*Lao1 (Iwanaga et al., 2004; Taviani et al., 2009; Marin et al., 2014). Nevertheless, structural variations, unstable core region, and the transfer region of both the ICEs found in our study were very much similar and shared a common ancestral backbone. In many ICEs, the core genes such as *int*, *bet, exo*, and *setR* are usually associated with phages, and genes such as *tra* are associated with plasmids (Wozniak et al., 2009; Armshaw and Pembroke, 2013). Having the same exclusion group (*eexR1*), ICE^GEN^ and ICE^TET^ were mutually exclusive and therefore did not co-exist in a strain. ICE sequences reconfirmed that there were two ICE types that kept emerging in different years. The key modifications between them indicated that they may have diverse origins or be derived from a common ancestor and could have later evolved independently.

We could transfer the ICE^GEN^ and ICE^TET^ from *V. cholerae* O1 to *E. coli* J53 by conjugation. The frequency of transfer observed was high (10^–5^ to 10^–6^), indicating that the ICEs were promiscuous due to the presence of an active *tra* region (Kiiru et al., 2009; Pande et al., 2012). Our study showed that only the resistances conferred by genes present in ICE were transferable and that the level of expression was different, being more in the transconjugants with respect to the donor vibrios. This could be due to “gene dosage” effect or absence of repressor in the new genetic environment of the recipient *E. coli*. Transconjugants showing higher drug resistance have been described in the previous reports as well (Petroni et al., 2002; Sarkar et al., 2015b). The co-existence of ICEs with plasmids and class 1 integrons in clinical as well as environmental *V. cholerae* has been reported (Thungapathra et al., 2002; Pande et al., 2012). The involvement of plasmids carrying the ICEs was not tested in this study. We also observed that resistance to NA and AMP were not transferable, indicating that the resistance to these antimicrobials could be contributed by the chromosomal factors such as mutations and efflux pumps (Ghosh and Ramamurthy, 2011).

As shown in the PFGE analysis, the clonal relatedness of *V. cholerae* strains isolated during different years corresponded with the MDR profiles. ICE integrase-negative strains isolated in 2008, 2011, and 2012 were found to cluster together (cluster A). *V. cholerae* O1 strains harboring either ICE^GEN^ or ICE^TET^ were also grouped in different clusters (B and C, respectively). A similar observation was made with the outbreak strains of *V. cholerae* O1 in Kenya (Kiiru et al., 2009).

In conclusion, our findings revealed the existence of two types of ICEs in *V. cholerae* O1 strains from Kolkata. The ICE^GEN^ that contained conserved backbone genes was most commonly detected in *V. cholerae* O1 circulating around Kolkata. Features of the Kolkata *V. cholerae* O1 strains with ICE carrying the Tet^R^ encoding genes are unique and the sequence of the ICE^TET^ had several variations from other sequenced ICEs. Also the ICE^TET^ harboring *V. cholerae* O1 strains reappeared after 4 years of disappearance in Kolkata. Unique PFGE clusters of *V. cholerae* O1 harboring different ICEs are linked with the AMR patterns. The primer pair designed in this study may be useful in the detection of ICEs carrying the *tet*. The transmission potential of ICEs identified in this study was very high, as evidenced from the conjugation assay. Therefore, the impact of ICE regulation and interactions between bacteria prevailing in the same ecological niches should be explored in detail. Emergence of new types of ICEs may pose challenges in the existing cholera management strategies.

## Author Contributions

AG, TR, and KO conceived and designed the experiments. AS, DM, and GC performed the experiments. KO contributed reagents, materials, and analysis tools. TR and AM analyzed the data. AS and TR wrote the manuscript. All authors discussed the results, and reviewed and commented on the manuscript.

## Conflict of Interest Statement

The authors declare that the research was conducted in the absence of any commercial or financial relationships that could be construed as a potential conflict of interest. The reviewer AB declared a past co-authorship with several of the authors, GC, AM, TR, and AG, to the handling Editor.

## Footnotes

1 http://db-mml.sjtu.edu.cn/ICEberg

2 http://www.cbcb.umd.edu/software/glimmer-mg

3 www.scigenom.com/CANoPI
